# Prevalence, Demographics, and Risk of Severe Acute COVID-19

**DOI:** 10.7759/cureus.18851

**Published:** 2021-10-18

**Authors:** Bala Munipalli, Dacre Knight, Ilana Logvinov, Abd Moain Abu Dabrh

**Affiliations:** 1 Internal Medicine, Mayo Clinic, Jacksonville, USA; 2 Anesthesiology, Mayo Clinic, Jacksonville, USA

**Keywords:** severe infection, sars-cov-2, infectious diseases, epidemiology, covid-19

## Abstract

Background: Our goal was to assess the demographics, risk factors, and hospital admission and length of stay (LOS) among patients with acute COVID-19 and to identify whether age, smoking status, race, risk factors, and sex significantly affect the severity of illness according to hospitalization or admission to the intensive care unit (ICU). Severity was defined as admission to the hospital or ICU.

Methods: This retrospective cohort chart review included patients who received care from March 13 to August 17, 2020, at a single academic medical center. Age, COVID-19 risk factors, sex, race, smoking history, and hospital LOS were analyzed with hospital admission and ICU admission. Categorical variables were summarized.

Results: The chart review assessed 1,697 adult patients with various degrees of severity of COVID-19 illness: 23 patients had been admitted to the hospital, and 7 had been admitted to the ICU. Older age and more COVID-19 risk factors, as defined by the Centers for Disease Control and Prevention, were significantly associated with hospital admissions, and longer LOS was statistically associated with ICU admission.

Conclusions: Severe COVID-19 infection was associated with older age and more risk factors. Current smoking status, sex, and race were not significantly different between hospitalized patients with severe COVID-19 infection who were admitted to the ICU and those who were not admitted to the ICU.

## Introduction

Coronaviruses, which belong to the family Coronaviridae, are single-stranded RNA viruses that infect humans and cause severe respiratory, gastrointestinal tract, hepatic, and neurologic diseases [1]. Coronavirus disease 2019 (COVID-19) is caused by severe acute respiratory coronavirus 2 (SARS-CoV-2) or novel coronavirus. Disease transmission occurs through large respiratory droplets and aerosolized fluids of affected individuals during coughing and sneezing [2]. Additionally, the virus has been found in stool and urine samples of people with infections [2]. The first reported cases occurred in December 2019 in Wuhan, China, and in March 2020, the World Health Organization declared a global pandemic [2].

Current evidence shows that most acute cases of COVID-19 are mild or asymptomatic, and people who have symptoms usually recover within 6 weeks, depending on their immune function and age [3,4]. The most common symptoms are fever, cough, dyspnea, fatigue, sputum production, anorexia, chest tightness, myalgia, diarrhea, headache, sore throat, dizziness, nausea, vomiting, abdominal pain, and hemoptysis [5,6]. Over 90% of patients present with more than one symptom [7]. In severe cases, disease progression can lead to pneumonia, acute respiratory distress syndrome, and acute respiratory failure resulting in organ failure and death [8].

Risk factors for severe or complicated COVID-19 infection include underlying cardiovascular or respiratory disease, immunocompromised state, sickle cell disease, malignancy, chronic kidney disease, diabetes, and obesity [9]. The Centers for Disease Control and Prevention (CDC) and the COVID-19-associated hospitalization surveillance network have reported that 89% of patients hospitalized with COVID-19 have had a preexisting condition [9].

The highest fatality rates are among patients older than 80 years [10], and one meta-analysis showed that risk factors for severe disease (resulting in intensive care unit [ICU] admission) include male sex and age older than 50 years [11]. One theory about the difference in severity between sexes is that the expression of angiotensin-converting enzyme 2 receptors, the primary receptors for viral entry into cells, may be higher in males [9].

Epidemiologic COVID-19 data that evaluate race and ethnicity of patients who require hospitalization show that, although the majority of patients are White, other groups at risk include Black, Hispanic, Asian, and American Indian/Alaskan Native [9]. The racial distribution in the US is 76% Whites, 13% Blacks, and 18% Hispanics, but data for patients with COVID-19 who required hospitalization show that while the majority of patients were White (56.5%), this group also included Blacks (33%) and Hispanics (8%) [9]. Although this is not an association, these percentages may signify that minority populations are at higher risk. The difference may be explained by the distribution of preexisting comorbidities (e.g., diabetes, hypertension, obesity, asthma, and heart disease) that increase the risk of contracting COVID-19 [9]. Further work is needed to explore the link between these factors.

COVID-19 infections tend to be more severe among people who smoke because their lungs are at greater risk for the virus to bind and enter [12]. The mortality rate among smokers is 80% higher than among nonsmokers, and the mortality rate for patients with preexisting chronic obstructive pulmonary disease (COPD) is four times higher than for those without COPD [13]. Additionally, compared to nonsmokers, smokers are 1.4 times more likely to have severe symptoms of COVID-19 and 2.4 times more likely to be admitted to an ICU and require mechanical ventilation or die [14].

The purpose of our study was to assess the disease prevalence and the demographics and risk factors among patients evaluated in an outpatient clinic who had a severe infection and required hospital or ICU admission. For this study, we defined severe infection as COVID-19 in patients who required hospitalization. We hypothesized that severe acute COVID-19 occurs in patients who have specific risk factors, and we wanted to describe the prevalence of severe COVID-19 and associations with age, sex, race, and comorbidities for this cohort. Additionally, as this disease has evolved, the risk factors have changed. Therefore, we conducted a chart review assessment on the vaccination status of patients hospitalized more recently with COVID-19.

## Materials and methods

The study was approved by the Mayo Clinic Institutional Review Board.

Study design and patients

We conducted a single-center retrospective chart review study at Mayo Clinic in Jacksonville, Florida (IRB # 20-004421, determined to be exempt from the requirement for IRB approval - 45 CFR 46.104d, category 4). Patients were identified through our institution’s COVID-19 Virtual Clinic (CVC), and contact information was obtained from the electronic health record. Eligibility criteria included the following: 1) positive test results for SARS-CoV-2 by reverse transcriptase-polymerase chain reaction; 2) age 18 years or older; and 3) evaluation in the CVC between March 13 and August 17, 2020. For the chart review assessment, data were collected from the institutional dashboard. Patient information was de-identified, and vaccination status was noted for patients hospitalized with COVID-19 between November 19, 2020 and July 14, 2021.

Outcomes of interest

The primary outcome was the quantified prevalence of COVID-19 risk factors in our population. For this purpose, the risk factors were those that the CDC recognized as comorbidities that were significantly associated with severe COVID-19 illness, such as cancer, cerebrovascular disease, chronic kidney disease, COPD, diabetes (types 1 and 2), heart conditions (such as heart failure, coronary artery disease, or cardiomyopathies), obesity (body mass index ≥ 30; calculated as weight in kilograms divided by height in meters squared), pregnancy, and smoking (including current and former smokers) [15].

Statistical analysis

Data on age, number of COVID-19 risk factors, and hospital length of stay (LOS) were extracted from the electronic health record and summarized as median (interquartile range) and mean (SD); categorical variables were summarized as frequency (percentage of the sample). Risk factors were compared between 1) patients who were admitted to the hospital and patients who were not and 2) patients who were admitted to the ICU and patients who were not. The Wilcoxon rank-sum test was used to evaluate differences in numerical variables and the Pearson chi-square or Fisher Exact test was used to evaluate the proportional differences in discrete variables. A linear regression model was performed to evaluate the association between risk factors and hospital LOS among patients who were admitted to the hospital. All tests were two-sided and p-values less than 0.05 were considered statistically significant. All statistical analyses used R version 3.6.2 (RStudio).

Data availability

Data are available upon request.

## Results

The demographic analysis was based on 1697 patients who were evaluated in the CVD and had positive COVID-19 test results for the selected period. Of these patients, 23 were hospitalized. Tables 1, 2 summarize demographic data for patients with positive test results who were admitted for hospitalization and those who were not admitted. Age and number of COVID-19 risk factors were significantly different between patients who were admitted and those who were not admitted: Older patients and patients with more risk factors were more likely to be admitted. Additionally, patients with comorbidities-congestive heart failure, chronic pulmonary disease, cerebrovascular disease, coronary atherosclerosis, pulmonary hypertension, and diabetes with or without complications, were also significantly more likely to be hospitalized. 

**Table 1:** Demographic data for patients who had positive COVID-19 test results according to hospital admission status Note: Wilcoxon rank-sum test for numerical variables; Chi-square test for categorical variables

	Not Admitted (N=1674)	Admitted (N=23)	Total (N=1697)	P-value
Age				<0.001
Median (Range)	42.0 (0.0, 99.0)	64.0 (40.0, 84.0)	43.0 (0.0, 99.0)	
Mean (SD)	43.3 (18.0)	63.5 (14.0)	43.5 (18.1)	
Sex				0.447
Female	786 (47.1%)	9 (39.1%)	795 (47.0%)	
Male	883 (52.9%)	14 (60.9%)	897 (53.0%)	
Race				0.149
Non-White	496 (29.6%)	10 (43.5%)	506 (29.8%)	
White	1178 (70.4%)	13 (56.5%)	1191 (70.2%)	
Ethnicity				0.943
Hispanic/Latino	135 (9.1%)	2 (8.7%)	137 (9.1%)	
Not Hispanic/Latino	1344 (90.9%)	21 (91.3%)	1365 (90.9%)	
COVID-19 no. risk factors				<0.001
Median (Range)	1.0 (0.0, 9.0)	4.0 (0.0, 6.0)	1.0 (0.0, 9.0)	
Mean (SD)	1.3 (1.5)	3.9 (1.6)	1.3 (1.5)	
Congestive heart failure				<0.001
N-Miss	1	0	1	
No	1598 (95.5%)	17 (73.9%)	1615 (95.2%)	
Yes	75 (4.5%)	6 (26.1%)	81 (4.8%)	
Chronic pulmonary disease				<0.001
N-Miss	1	0	1	
No	1499 (89.6%)	12 (52.2%)	1511 (89.1%)	
Yes	174 (10.4%)	11 (47.8%)	185 (10.9%)	
Cerebrovascular disease				<0.001
N-Miss	1	0	1	
No	1615 (96.5%)	18 (78.3%)	1633 (96.3%)	
Yes	58 (3.5%)	5 (21.7%)	63 (3.7%)	
Coronary atherosclerosis				0.031
No	1639 (97.9%)	21 (91.3%)	1660 (97.8%)	
Yes	35 (2.1%)	2 (8.7%)	37 (2.2%)	
Pulmonary hypertension				<0.001
No	1654 (98.8%)	19 (82.6%)	1673 (98.6%)	
Yes	20 (1.2%)	4 (17.4%)	24 (1.4%)	
Diabetes with complications				<0.001
N-Miss	1	0	1	
No	1609 (96.2%)	17 (73.9%)	1626 (95.9%)	
Yes	64 (3.8%)	6 (26.1%)	70 (4.1%)	
Diabetes without complications				<0.001
N-Miss	1	0	1	
No	1510 (90.3%)	14 (60.9%)	1524 (89.9%)	
Yes	163 (9.7%)	9 (39.1%)	172 (10.1%)	

**Table 2:** Supplementary to Table 1: Frequency table for the race by ethnicity

	Hispanic/Latino (N=137)	Not Hispanic or Latino (N=1,365)	Unknown (N=195)	Total (N=1,697)	P-value
Race					<0.001
American Indian or Alaska Native	0 (0.0%)	13 (1.0%)	0 (0.0%)	13 (0.8%)	
Asian	0 (0.0%)	35 (2.6%)	2 (1.0%)	37 (2.2%)	
Black or African American	7 (5.1%)	226 (16.6%)	1 (0.5%)	234 (13.8%)	
Other or Unknown	34 (24.8%)	18 (1.3%)	170 (87.2%)	222 (13.1%)	
White	96 (70.1%)	1,073 (78.6%)	22 (11.3%)	1,191 (70.2%)	

Hospital LOS was significantly longer among patients who were admitted to the ICU compared with those who were not (Table 3). Age, sex, race, ethnicity, the number of COVID-19 risk factors, smoking status, and comorbidities were not significantly associated with ICU admission. Table 4 shows the results of a univariable linear regression model predicting hospital LOS based on the subset of patients admitted. The patients with ICU admission stayed in the hospital significantly longer. White patients were likely to have a shorter hospital stay than non-white patients.

**Table 3:** Demographic and clinical data for hospitalized patients according to ICU admission status Note: Wilcoxon rank-sum test for numerical variables; Fisher Exact test for categorical variables LOS - length of stay

	No-ICU (N=16)	Yes-ICU (N=7)	Total (N=23)	P-value
Age				0.422
Median (Range)	66.0 (40.0, 84.0)	59.0 (44.0, 84.0)	64.0 (40.0, 84.0)	
Mean (SD)	64.9 (13.6)	60.3 (15.4)	63.5 (14.0)	
Sex				1.000
Female	6 (37.5%)	3 (42.9%)	9 (39.1%)	
Male	10 (62.5%)	4 (57.1%)	14 (60.9%)	
Race				0.169
Non-White	5 (31.2%)	5 (71.4%)	10 (43.5%)	
White	11 (68.8%)	2 (28.6%)	13 (56.5%)	
Ethnicity				0.158
Asian	2 (12.5%)	2 (28.6%)	4 (17.4%)	
Black or African American	2 (12.5%)	3 (42.9%)	5 (21.7%)	
Other or Unknown	1 (6.2%)	0 (0.0%)	1 (4.3%)	
White	11 (68.8%)	2 (28.6%)	13 (56.5%)	
COVID-19 no. risk factors				0.412
Median (Range)	4.5 (0.0, 6.0)	3.0 (2.0, 6.0)	4.0 (0.0, 6.0)	
Mean (SD)	4.0 (1.7)	3.6 (1.5)	3.9 (1.6)	
Smoking				1.000
Non-Smoker	10 (62.5%)	5 (71.4%)	15 (65.2%)	
Smoker	6 (37.5%)	2 (28.6%)	8 (34.8%)	
Congestive heart failure				0.621
No	11 (68.8%)	6 (85.7%)	17 (73.9%)	
Yes	5 (31.2%)	1 (14.3%)	6 (26.1%)	
Chronic pulmonary disease				0.193
No	10 (62.5%)	2 (28.6%)	12 (52.2%)	
Yes	6 (37.5%)	5 (71.4%)	11 (47.8%)	
Cerebrovascular disease				0.272
No	11 (68.8%)	7 (100.0%)	18 (78.3%)	
Yes	5 (31.2%)	0 (0.0%)	5 (21.7%)	
Coronary atherosclerosis				1.000
No	14 (87.5%)	7 (100.0%)	21 (91.3%)	
Yes	2 (12.5%)	0 (0.0%)	2 (8.7%)	
Pulmonary hypertension				1.000
No	13 (81.2%)	6 (85.7%)	19 (82.6%)	
Yes	3 (18.8%)	1 (14.3%)	4 (17.4%)	
Diabetes with complications				0.621
No	11 (68.8%)	6 (85.7%)	17 (73.9%)	
Yes	5 (31.2%)	1 (14.3%)	6 (26.1%)	
Diabetes without complications				0.363
No	11 (68.8%)	3 (42.9%)	14 (60.9%)	
Yes	5 (31.2%)	4 (57.1%)	9 (39.1%)	
Hospital LOS				< 0.001
Median (Range)	5.5 (1.0, 20.0)	20.0 (10.0, 59.0)	8.0 (1.0, 59.0)	
Mean (SD)	6.9 (4.8)	30.3 (20.4)	14.0 (15.8)	

**Table 4:** Univariable linear regression model for prediction of hospital LOS among patients admitted to ICU

Variables	Estimate	CI95.lower	CI95.upper	P-value
Age	-0.22	-0.73	0.28	0.367
Sex (Male)	2.26	-12.07	16.60	0.746
Race (White)	-13.19	-26.01	-0.37	0.044
Ethnicity (Not Hispanic/Latino)	6.62	-18.09	31.33	0.583
COVID-19	-0.68	-5.06	3.70	0.750
ICU admission (Yes-ICU)	23.35	12.39	34.31	0.000
Smoking (Smoker)	-2.75	-17.42	11.92	0.701
Congestive heart failure (Yes)	-5.02	-20.83	10.79	0.516
Chronic pulmonary disease (Yes)	8.11	-5.45	21.66	0.227
Cerebrovascular disease (Yes)	-8.74	-25.28	7.79	0.284
Coronary atherosclerosis (Yes)	-1.69	-26.57	23.19	0.889
Pulmonary hypertension (Yes)	-7.92	-26.07	10.23	0.374
Diabetes with complications (Yes)	-7.05	-22.70	8.60	0.360
Diabetes without complications (Yes)	4.86	-9.35	19.06	0.485

Among hospitalized patients who were not admitted to the ICU, the median age was 66.0 years and the mean LOS was 6.9 days, 31.2% were not White, 12.5% were Hispanic/Latino, 62.5% were male, and 37.5% were smokers. Among hospitalized patients who were admitted to the ICU, the mean age was 60.3 years, 71.4% were not White (none were Hispanic/Latino however), and the mean LOS was 30.3 days. Age, sex, ethnicity, number of COVID-19 risk factors, and smoking status showed no significant association with ICU admission and increased LOS (Table 4). Patients admitted to the ICU were more likely to have a significantly longer LOS than patients not admitted to the ICU, and patients who were White were more likely to have a shorter hospital stay than patients who were not White.

Compared to our data from more than one year previously, current outcomes of severe COVID-19 can be influenced by vaccination status. Of 573 inpatients from November 19, 2020 to July 14, 2021, a total of 142 patients had been vaccinated compared with 75% of patients hospitalized with COVID-19 who had not been vaccinated.

## Discussion

The results of our study showed a significant correlation between older age, comorbidity, and hospital admission and between hospital LOS and ICU admission. ICU admission was not significantly correlated with age, sex, race, comorbidity, or tobacco use.

Limitations of the study include lack of ethnic diversity: 28% to 70.5% of deaths from COVID-19 occur in Blacks, and the infection risk is three times higher in Black communities compared with predominantly White communities [16]. The likelihood of hospitalization or death from COVID-19 is four to five times higher among non-Hispanic Native Americans or Alaska Natives, non-Hispanic Blacks, and Hispanic or Latino persons than among non-Hispanic White persons [17,18]. Repeating our study with a population that had more ethnic diversity would be helpful, especially because preexisting conditions are well-recognized factors for COVID-19 and the incidence of preexisting conditions varies among ethnic groups.

Additionally, studies have shown that women are less susceptible to viral infection than men, possibly because the X chromosome and sex hormones provide protection and affect both innate and adaptive immunity [18]. Since men are more likely to smoke and to have underlying conditions, it may be useful to consider how sex affects prognosis in the face of underlying risk factors. CDC data from China showed that men had a higher mortality rate than women, but a higher percentage of men than women were smokers (52.9% vs 2.4%), so further research would likely be required to determine how sex and underlying conditions affect prognosis [19].

Respiratory failure is the leading cause of death among patients with COVID-19 because of acute respiratory disease syndrome [20], so patients who vape or smoke tobacco or marijuana could have an especially increased risk of life-threatening complications due to the damaging effects on the lungs. Opioid use disorder and misuse of methamphetamines can also have damaging effects on the respiratory system and may increase the risk of life-threatening complications. In addition, people who misuse substances are more likely to experience homelessness and incarceration than others, so they may have an increased risk for infection [20]. This factor must be considered when outpatient care for this population is addressed. Another limitation to the study is regarding vaccination and hospitalization; specifically, the clinical condition of patients who are vaccinated and hospitalized. In our experience, these have mostly been patients with comorbid conditions (late-stage cancer, end-stage respiratory disease, organ failure from other processes, immunosuppression from an organ transplant, etc.) that make one at high risk for severe disease already. We do not have the data available to confirm this hypothesis. That will need to be tested further in future work.

Other prognostic factors associated with disease progression include increased levels of interleukin-6, hypoalbuminemia, hyperglycemia, thrombocytopenia, lymphopenia, leukocytosis, high ratio of a number of neutrophils to a number of lymphocytes, and liver or kidney impairment [19]. The ability to distinguish the effects of each condition among patients would be beneficial for further developing tools to better understand the prognosis for outpatients with COVID-19.

Upon review of conditions and risk factors for severe COVID-19, we would be remiss to not consider vaccination status. This has been of burgeoning interest worldwide. As we have demonstrated here, vaccination status is an important characteristic of patients with severe COVID-19. Vaccine breakthrough and COVID-19 variant virulence resulting in hospitalization also need to be explored. It will be the prerogative of policymakers and health care leaders worldwide to encourage continued vaccination for COVID-19 as it becomes a more widely preventable condition.

## Conclusions

Our findings describe the prevalence of severe COVID-19 in our sample of patients. Older age and a larger number of risk factors for COVID-19 were associated with hospital admission, and ICU admission and White race were significant determinants of LOS. Lack of vaccination is emerging as an additional risk factor for severe infection. Further research should lead to improved screening tools and guide the necessary steps in evaluation and clinically effective treatment. Our study provides a framework to support early recognition and treatment of patients at increased risk for severe COVID-19. It also highlights risk factors such as demographic characteristics that increase the risk of severe acute COVID-19.

## References

[1] Wu D, Wu T, Liu Q, Yang Z (2020). The SARS-CoV-2 outbreak: what we know. Int J Infect Dis.

[2] Karia R, Gupta I, Khandait H, Yadav A, Yadav A (2020). COVID-19 and its modes of transmission. SN Compr Clin Med.

[3] Yang X, Yu Y, Xu J (2020). Clinical course and outcomes of critically ill patients with SARS-CoV-2 pneumonia in Wuhan, China: a single-centered, retrospective, observational study. Lancet Respir Med.

[4] Knight D, Downes K, Munipalli B, Halkar MG, Logvinov II, Speicher LL, Hines SL (2021). Symptoms and clinical outcomes of coronavirus disease 2019 in the outpatient setting. SN Compr Clin Med.

[5] Qian GQ, Yang NB, Ding F (2020). Epidemiologic and clinical characteristics of 91 hospitalized patients with COVID-19 in Zhejiang, China: a retrospective, multi-centre case series. QJM.

[6] Xu XW, Wu XX, Jiang XG (2020). Clinical findings in a group of patients infected with the 2019 novel coronavirus (SARS-Cov-2) outside of Wuhan, China: retrospective case series. BMJ.

[7] Wang R, Pan M, Zhang X (2020). Epidemiological and clinical features of 125 hospitalized patients with COVID-19 in Fuyang, Anhui, China. Int J Infect Dis.

[8] Branley JM, Polkinghorne A, Gilbert GL (2020). Reusing N95 (or P2) masks: current evidence and urgent research questions. Med J Aust.

[9] Garg S, Kim L, Whitaker M (2020). Hospitalization rates and characteristics of patients hospitalized with laboratory-confirmed coronavirus disease 2019 - COVID-NET, 14 States, March 1-30, 2020. MMWR Morb Mortal Wkly Rep.

[10] Bonanad C, García-Blas S, Tarazona-Santabalbina F (2020). The effect of age on mortality in patients with COVID-19: a meta-analysis with 611,583 subjects. J Am Med Dir Assoc.

[11] Barek MA, Aziz MA, Islam MS (2020). Impact of age, sex, comorbidities and clinical symptoms on the severity of COVID-19 cases: a meta-analysis with 55 studies and 10014 cases. Heliyon.

[12] Gupta AK, Nethan ST, Mehrotra R (2021). Tobacco use as a well-recognized cause of severe COVID-19 manifestations. Respir Med.

[13] Zhao Q, Meng M, Kumar R (2020). The impact of COPD and smoking history on the severity of COVID-19: a systemic review and meta-analysis. J Med Virol.

[14] Vardavas CI, Nikitara K (2020). COVID-19 and smoking: a systematic review of the evidence. Tob Induc Dis.

[15] Centers for Disease Control and Prevention (2021). Underlying medical conditions associated with high risk for severe COVID-19: information for healthcare providers. https://www.cdc.gov/coronavirus/2019-ncov/hcp/clinical-care/underlyingconditions.html.

[16] Yancy CW (2020). COVID-19 and African Americans. JAMA.

[17] Khunti K, Singh AK, Pareek M, Hanif W (2020). Is ethnicity linked to incidence or outcomes of covid-19?. BMJ.

[18] Gal-Oz ST, Maier B, Yoshida H (2019). ImmGen report: sexual dimorphism in the immune system transcriptome. Nat Commun.

[19] Noh JY, Yoon JG, Seong H (2020). Asymptomatic infection and atypical manifestations of COVID-19: comparison of viral shedding duration. J Infect.

[20] Ruan Q, Yang K, Wang W, Jiang L, Song J (2020). Clinical predictors of mortality due to COVID-19 based on an analysis of data of 150 patients from Wuhan, China. Intensive Care Med.

